# Nutritional Modulation of Hepcidin in the Treatment of Various Anemic States

**DOI:** 10.3390/nu15245081

**Published:** 2023-12-12

**Authors:** Patrizia D’Andrea, Francesca Giampieri, Maurizio Battino

**Affiliations:** 1 Department of Clinical Sciences, Polytechnic University of Marche, 60131 Ancona, Italy; f.giampieri@univpm.it; 2 Research Group on Foods, Nutritional Biochemistry and Health, Universidad Europea del Atlántico, Isabel Torres 21, 39011 Santander, Spain; costaborea@libero.it; 3 International Joint Research Laboratory of Intelligent Agriculture and Agri-Products Processing, Jiangsu University, Zhenjiang 212013, China

**Keywords:** hepcidin, iron homeostasis, ferroptosis, anemia

## Abstract

Twenty years after its discovery, hepcidin is still considered the main regulator of iron homeostasis in humans. The increase in hepcidin expression drastically blocks the flow of iron, which can come from one’s diet, from iron stores, and from erythrophagocytosis. Many anemic conditions are caused by non-physiologic increases in hepcidin. The sequestration of iron in the intestine and in other tissues poses worrying premises in view of discoveries about the mechanisms of ferroptosis. The nutritional treatment of these anemic states cannot ignore the nutritional modulation of hepcidin, in addition to the bioavailability of iron. This work aims to describe and summarize the few findings about the role of hepcidin in anemic diseases and ferroptosis, as well as the modulation of hepcidin levels by diet and nutrients.

## 1. Introduction

Hepcidin is a peptide hormone, consisting of 25 amino acids in the bioactive form with a molecular weight of 2.8 KD [1,2]. It is mainly synthesized by the liver and excreted by the kidneys; under physiological conditions, it reaches plasma concentrations between 2 and 20 nM [1,2,3,4].

The receptor of hepcidin is ferroportin, which consists of 571 amino acids and has a molecular weight around 65–70 KD. It is a channel that permits the efflux of elemental iron from cells to the plasma and is formed by two transmembrane domains (each with six alpha-helices) and a lysine-rich cytoplasmic loop [2,5,6]. Ferroportin is expressed on the basolateral membrane of duodenal enterocytes, in macrophages responsible for erythrophagocytosis (i.e., Kupffer cells and red pulp cells of the spleen) in hepatocytes and other cells of iron-store tissues [7,8]. The binding of hepcidin to ferroportin blocks the flow of iron by both a short-term and a long-term mechanism. In the first case the ligand generates an immediate steric hindrance, closing the open conformation of the channel [7,9]. In the second case, hepcidin triggers endocytosis and ubiquitination in the lysine residues of the cytosolic loop by Rnf217 ligase E3, resulting in channel degradation [2,8]. Recently, a novel mechanism has been identified for Rnf217, considered as a E3 ubiquitin ligase, which is able to mediate the degradation of the iron exporter ferroportin and regulate iron homeostasis [10].

Since ferroportin is the only known cellular iron export system, plasma hepcidin fluctuations regulate the actual amount of dietary iron absorbed, released from stores, present in the recycling system, and made available to erythropoiesis [2,6].

The aim of this work is to summarize the few recent findings about the role exerted by hepcidin on anemic diseases and ferroptosis, as well as the modulation of hepcidin levels by diet and nutrients.

## 2. Regulation of Hepcidin Release

**Figure 1:**
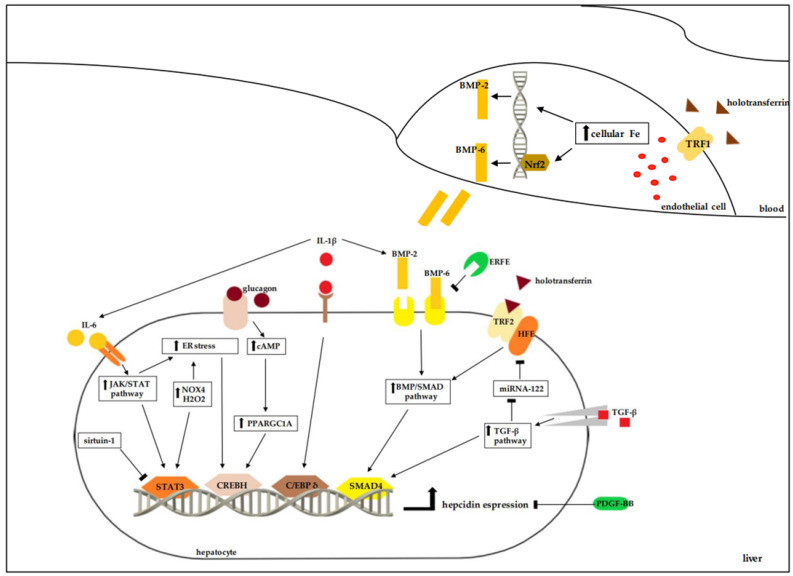
Main mechanisms involved in the regulation of hepcidin release. Increased iron accumulation in the liver stimulates hepcidin expression through two mechanisms, which converge during the activation of the SMAD4 factor. SMAD4 is the last player of the BMP/SMAD pathway, activated both by the increase in cellular iron in endothelial cells, which releases BMP-2 and BMP-6, and by the formation of the HFE-TRF2 complex in hepatocytes, after binding to holotransferrin. Inflammation, with its cytokines, can increase expression of hepcidin through various transcription factors: TGF-β acts on SMAD4; IL-1β stimulates C/EBP δ; and IL-6 actives STAT3. Even mild or moderate hypoxia, with increased NOX4 activity, can increase hepcidin release by activating STAT3. Increase in NOX4 activity acts on reticular stress, which stimulates hepcidin synthesis with the activation of the CREBH factor. The same factor is also involved in the metabolic responses induced by fasting. Finally, other molecules such as ERFE, sirtuin-1, miR-NA-122 and PDGF-BB, inhibit the activation of these transcription factors to varying degrees, reducing the expression of hepcidin. BMP/SMAD, Bone morphogenetic protein/Small Mother Against Decapentaplegic; BMP-2, Bone morphogenetic protein 2; BMP-6, Bone morphogenetic protein 6; cAMP, Cyclic Adenosine Mono Phosphate; C/EBP δ, CCAAT Enhancer-binding protein δ; CREBH, Cyclic AMP-responsive element-binding protein H; ER, Endoplasmic Reticulum; ERFE, Erythroferrone; Fe, iron; HFE, Hereditary hemochromatosis protein; IL-1β, Interleukin 1 β; IL-6, Interleukin 6; JAK/STAT, Janus kinase/Signal Transducer and Activator of Transcription; miRNA-122, micro Ribonucleic Acid 122; NOX4, NADPH Oxidase 4; Nrf2, Nuclear factor erythroid 2-related factor 2; PDGF-BB, Platelet-derived growth factor-BB; PPARGC1A, Peroxisome Proliferator-Activated Receptor Gamma Coactivator 1 α; SMAD4, Small Mother Against Decapentaplegic 4; STAT3, Signal Transducer and Activator of Transcription 3; TGF-β, Tumor growth factor β; TRF1, transferrin receptor 1; TRF2, transferrin receptor 2.

Hepcidin release is crucial for maintaining iron homeostasis and is controlled by several mechanisms (Figure 1).

Two conditions mainly regulate the increase in plasma hepcidin levels: the iron status of the body and the inflammatory process. On one side, the release of hepcidin from the liver rises in response to increasing iron content in the body [11]. The liver can detect, through two distinct systems, both the amount of iron deposited and the plasma iron peaks [11]. Increased iron deposition in hepatic endothelial cells activates the Nuclear factor erythroid 2-related factor 2 (Nrf2), which results in the expression of the paracrine signaling molecules, such as Bone morphogenetic protein 2 (BMP2) and Bone morphogenetic protein 6 (BMP6) [12,13,14]. In particular, activation of the Nrf2, following an increase in cellular iron, stimulates BMP6 synthesis [15]. In turn, in hepatocytes, BMP2 and BMP6 activate the Bone morphogenetic protein-Small Mother Against Decapentaplegic (BMP/SMAD) pathway in hepatocytes, which induces the expression of the hepcidin gene [11,16]. The increase in plasma iron, through the increase in holotransferrin, is captured in hepatocytes with the activation of the homeostatic iron regulator—transferrin receptor 2 (HFE-TRF2) complex [17]. Homeostatic iron regulator (HFE) is a membrane protein complex expressed in hepatocytes [18]; it can bind at different sites, including both Transferrin receptor 1 (TFR1) (ubiquitous high-affinity holotransferrin receptor) and Transferrin receptor 2 (TFR2) (low-affinity holotransferrin receptor expressed predominantly in hepatocytes) [18,19,20,21]. Holotransferrin competes with HFE for binding to TRF1 and simultaneously stabilizes the bond between HFE and TRF2 [21]. Consequently, an increase in plasma holotransferrin causes the formation of the HFE-TRF2 complex, which activates the SMAD pathway [22]. Both systems, therefore, converge in the activation of the BMP/SMAD pathway, which involves numerous molecules. The terminal effector is the transcription factor Small Mother Against Decapentaplegic 4 (SMAD4), which promotes the expression of the hepcidin gene [11]. SMAD4 also acts epigenetically, allowing for the methylation of lysine 4 in histone H3, which opens chromatin and leads to transcription [23]. This epigenetic effect renders SMAD4 also necessary in the stimulation of inflammation-mediated hepcidin expression [11,23]. Therefore, the increase in hepcidin following inflammation may be limited in the absence of iron.

On the other side, the inflammatory process involves an increase in hepcidin levels [24], following an immune protection mechanism implemented by hypoferremia. Interleukin (IL) 6 is mainly responsible for the increase in hepcidin expression, through the activation of the transcription factor Signal Transducer and Activator of Transcription 3 (STAT3) [7,25]. Other inflammatory cytokines are also involved, such as IL-1β, IL-22, and Tumor growth factor-β (TGF-β) [7]. IL-1β activates the transcriptional factor CCAAT Enhancer-binding protein δ (C/EBP δ), which increases the expression of hepcidin and amplifies the activation of STAT3 and SMAD4 factors, thus stimulating the release of IL-6 and BMP2 in hepatocytes [26]. Similarly, the TGF- β pathway involves the activation of SMAD proteins, including SMAD 4 [27]. Inflammation is one factor that can trigger the Endoplasmic Reticulum (ER) stress response. ER stress increases hepcidin expression through an independent pathway that activates Cyclic AMP-responsive element-binding protein H (CREBH) factor [28]. Furthermore, a study conducted on mice demonstrated that fasting-induced gluconeogenesis increases hepcidin levels precisely by stimulating CREBH/Peroxisome Proliferator-Activated Receptor Gamma Coactivator 1 α (PPARGC1A) factors [29].

Hepcidin expression is also regulated by microRNAs. The main known candidate is miRNA-122, expressed mainly in the liver [30]. In mice, miRNA-122 decreases hepcidin release, silencing the HFE and Hemojuvelin protein genes involved in SMAD4 activation [31]. A recent study conducted on mouse and human models showed that TGF-β1 drastically reduces the levels of miRNA-122 in the liver through activation of the SMAD pathway [32]. The decrease in miRNA-122 contributes to the increase in hepcidin release during the inflammatory process. However, in some pathological states, such as cardiovascular fibrosis, miRNA-122 stimulates the TGF- β pathway [33], which increases hepcidin release [27]. Further studies are necessary to clarify the role of miRNA-122 in relation to hepcidin.

Other pathways are also involved in hepcidin expression. Sirtuin-1 is a deacetylase implicated in the control of inflammation and oxidative stress [34]. It acts on STAT3 by blocking the induction of hepcidin [35]. The erythropoiesis process reduces hepcidin levels by increasing iron absorption. Many factors produced by erythrocytes are implicated, such as Growth differentiation factor 15, Twisted gastrulation [36], and, in particular, Erythropoietin (EPO), which promotes the synthesis of Erythroferrone (ERFE), which binds to BMP-6 and switches off BMP-SMAD signaling [37]. The role of hypoxia is more controversial. Chronic hypoxia, such as that which occurs at high altitude, down-regulates the hepcidin gene by platelet-derived growth factor-BB (PDGF-BB) [38]. Conversely, mild or pronounced hypoxia increases the release of hepcidin due to increased activity of oxidases such as NADPH Oxidase 4 (NOX4). The major product of NOX4 is hydrogen peroxide, which can activate the STAT3 factor [39]. Finally, a study demonstrated the ability of Interferon-γ to reduce hepcidin expression in macrophages infected with siderophilic bacteria [40].

## 3. Anemic States Characterized by the Increase in Hepcidin Release

Various conditions can favor an increased release of hepcidin and can cause a state of anemia. For example, inflammatory anemia (IA) is associated with pathological conditions, such as chronic inflammatory diseases, infections and malignant tumors. In these diseases, the inflammatory pathway increases hepcidin levels. IA is a mild to moderate anemia, usually normocytic, with low serum iron and transferrin, non-low plasma ferritin, and hemoglobin rarely less than 8 mg/dL [41]. At the same time, senile anemia can in some cases be an anemic condition caused by the increase in hepcidin. A European study showed that senile anemia in hospital patients is more strongly associated with inflammatory conditions and renal failure, rather than nutritional deficiencies [42]. Multiple factors can increase hepcidin in the elderly: the stimulation of the inflammatory pathway in chronic diseases or infections, the reduced production of EPO due to increased age [43] or due to renal disease [44], and the decreased clearance of hepcidin in chronic renal failure [45].

Another type of anemia, characterized by increased hepcidin, is associated with obesity. The correlation between anemia and obesity has been widely discussed, and several epidemiological studies have confirmed this association [46,47,48], although, obesity and anemia were not correlated in Chinese women [49] and in children in Tajikistan [50]. However, the peculiarity of the social and food context in which these surveys were conducted could partly explain the discrepancy in the results.

Obesity generates low-grade systemic inflammation with an increase in IL-6 [51]. Increasing leptin in individuals with obesity may also stimulate hepcidin release [52]. In fact, hepcidin is higher in individuals with obesity than in individuals with normal weight [53,54]. Nonetheless, a study conducted on children with obesity demonstrated that the increase in hepcidin is not accompanied by a significant increase in IL-6, underestimating the role of hepcidin in anemia [55]. Other factors can influence hepcidin level in obesity, such as IL-10, IL-1β and miRNA-122. In adolescents with obesity, the decrease in IL-10 and the increase in IL-1β [56] stimulate hepcidin release, while in childhood obesity, the increase in miRNA-122 [57,58] can limit hepcidin levels [31].

Increased hepcidin plays a role also in diabetes-associated anemia. Clinical practice demonstrated improvements in anemia in type 2 diabetes following the administration of the Sodium–glucose cotransporter 2 inhibitor [59]. This drug decreased hepcidin both by increasing EPO levels [60] and by stimulating the expression of sirtuin-1 [61]. Increased hepcidin in type 2 diabetes is related to nephropathy [44,45,62] but not alone. Insulin resistance and prolonged gluconeogenesis in diabetes activate CREBH [63], which stimulates hepcidin release [29].

Finally, many studies reported iron deficiency as a frequent condition in both professional [64,65] and non-professional [66] athletes, especially female ones. If left untreated, iron deficiency can develop into anemia. Other factors can cause anemia in athletes, such as increased plasma volume, increased body temperature, acidosis, gastrointestinal bleeding and increased hemolysis [67], although one study debunked the role of foot-strike hemolysis in the anemia of marathon runners [68]. Despite this, iron deficiency is the only modifiable factor to prevent sports anemia. Hepcidin plays a role in athletes’ anemia. Many studies have reported the increase in hepcidin following high or moderate intensity physical exercises [69]. In general, hepcidin levels peak 3 h after exercise, and return to baseline levels after 6 h [69]. Multiple factors modulate hepcidin release in physical activity. IL-6 increases in response to exercise [70,71,72] and stimulates hepcidin release [7,25]. Conversely, low serum ferritin limits hepcidin release [72,73]. Additionally, the type of physical activity performed can influence the release of hepcidin through the production of oxidative stress, both through the role of free radicals on STAT3 [39] and with the induction of ER stress [28]. Intense and prolonged exercise increases oxidative stress [74] and favors the increase in hepcidin. On the other hand, regular and moderate physical activity reduces the production of free radicals [75]. In sports, hepcidin can increase due to the activation of the CREBH pathway [29]. A recent study conducted on mice demonstrated how the depletion of glucose reserves during physical activity stimulates the release of hepcidin, through gluconeogenetic signals [76]. The increase in hepcidin levels after sporting activity can reduce the availability of iron introduced by diet or supplements, and promote both iron deficiency and anemia.

## 4. Ferroptosis and Hepcidin

Ferroptosis is a form of autophagic cell death [77], involved in numerous pathological states, such as tumors, neurodegenerative diseases and reperfusion injuries [78]. Ferroptosis is characterized by iron-mediated lipid peroxidation and by the inefficiency of reduced glutathione and glutathione peroxidase 4 (GPX4). The cellular labile iron pool is crucial in the mechanism of ferroptosis. Free iron produces radicals through Fenton reactions and composes enzymes involved in lipid oxidation, such as Arachidonate lipoxygenase, NADPH Oxidase and Cytochrome P450 [79]. Under physiological conditions, hepcidin limits iron accumulation in the body and this should protect against ferroptosis. However, an uncontrolled increase in hepcidin can contribute to an increase in cellular labile iron. Hepcidin acts mainly on ferroportin, which is involved in the ferroptosis mechanism. A study conducted on neuroblastoma cells treated with erastin demonstrated that the expression of ferroportin acted on ferroptosis, modifying the concentration of cellular iron. The expression of ferroportin negatively modulated ferroptosis in cells, while the knockdown of ferroportin enhanced the antitumor activity of erastin by increasing cellular iron [80]. In addition, failure to express ferroportin in the brain in mice led to the development of an Alzheimer’s-like phenotype. The increase in ferroptosis led to the loss of memory and neurons. Instead, increasing ferroportin expression reduces ferroptosis and improves memory [81]. Similarly, the deubiquitinase USP35 stabilized ferroportin and blocked ferroptosis in lung cancer cells [82]. In contrast, miRNA 302-a 3-p induced ferroptosis in human non-small cell lung cancer cells by inhibiting ferroportin expression [83]. Thus, ferroptosis is promoted by the repression of ferroportin, which is the main action mediated by hepcidin. However, ferroportin is not the only link between hepcidin and ferroptosis. A study performed on mouse models that simulate early damage after sub-arachnoid hemorrhage, demonstrated that the increase in hepcidin stimulates the expression of the Divalent metal transporter 1 (DMT1). The increase in DMT1, and the decrease in ferroportin, increases cellular iron and promotes ferroptosis [84].

**Figure 2:**
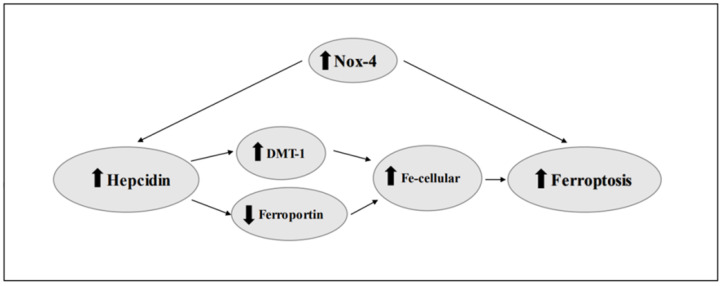
Link between hepcidin and ferroptosis. DMT1, Divalent metal transporter 1; NOX4, NADPH Oxidase 4.

NOX4 Oxidase is another link between hepcidin and ferroptosis (Figure 2). Studies on differentially expressed genes (DEGs) reported that the NOX4 gene is strongly related to ferroptosis in colorectal cancer [85] and in gastric adenocarcinoma [86]. The increase in the expression and activity of NOX4 increases oxidative stress [87], and this promotes lipid peroxidation. However, in ferroptosis, lipid peroxidation is mediated by iron. Not surprisingly, NOX4 induces the increase in hepcidin [39], and therefore increases the amount of cellular iron. It is plausible to think that the strong correlation between the expression of NOX4 and ferroptosis is due to the increase in cellular iron, ensured precisely by the increase in hepcidin levels (Figure 2).

## 5. Nutritional Modulation of Hepcidin

Classical nutritional treatment for anemia involves increasing iron intake by enhancing dietary bioavailability and intake [88]. Foods rich in polyphenols, phytic acid, oxalic acid and calcium are not recommended, as they reduce the intestinal absorption of iron. Instead, foods that increase the intestinal absorption of iron, such as animal tissues, including chicken, beef, pork, lamb and fish, and ascorbic acid, are recommended [88]. In anemias characterized by hepcidin elevation, except in sports anemia, the iron reserve in the body is often already abundant [41]. In these anemic states promoting iron absorption exclusively, without considering hepcidin, can be ineffective or even dangerous in view of the link with ferroptosis. On the contrary, knowing the nutritional modulation of hepcidin may be beneficial. Some foods and nutrients have a known or potential ability to influence hepcidin release, as shown in Table 1.

For example, omega-3 fatty acids, in particular Eicosapentaenoic acid (EPA), Decosahexaenoic acid (DHA) and their derivatives, are able to suppress the inflammatory process through multiple mechanisms, including the reduction in the expression of inflammatory cytokines [118,119]. Indeed, a recent study demonstrated the improvement of anemia in mice infected with tuberculosis following the uncombined administration of Fe and EPA/DHA for 3 weeks and a reduction in IL-6 and IL-1 levels [89], both involved in the synthesis of hepcidin [7], even if a direct estimate of hepcidin in mice before and after correction of anemia is lacking.

Regarding polyphenol-containing foods, coffee consumption is usually limited in the diets of anemics. In fact, coffee polyphenols reduce the absorption of iron [88]. However, in conditions characterized by an increase in hepcidin, coffee can improve anemia. In this context, in a study conducted on mice treated with the daily intragastric administration of caffeine for 7 days and then injected with lipopolysaccharide (LPS), caffeine suppressed hepatic hepcidin expression and diminished its overexpression caused by LPS by inhibiting the IL-6/STAT3 pathway [90]. Similarly, red wine, due to its high content of polyphenols, is usually not recommended in the diet of anemic people [88]. Yet, the moderate consumption of red wine for 3 weeks reduced the expression of hepcidin in healthy subjects and those with type 2 diabetes [91], improving iron trafficking. Tucum-do-cerrado (*Bactris setosa* Mart.) is another food rich in polyphenols typical of the Brazilian diet. In rats supplemented with a tucum-do-cerrado-enriched diet for 12 weeks, this fruit has shown to reduce hepcidin levels through multiple mechanisms, including increasing the activity of sirtuin-1 [92]. Similarly, dark leafy vegetables, also abundant in polyphenols, are normally recommended for anemics as a source of folic acid and since they exert an iron-inhibitory effect, probably due to the phenol- or phytate-mediated iron-chelating activity [120]. However, according to a frequency study, performed on pregnant women, the high consumption of green leafy vegetables increased hepcidin levels [93].

Malted oats are recommended in anemia due to the greater availability of iron [121], yet habitual consumption of oats is associated with greater release of hepcidin [93]. This cereal contains isoflavones, polyphenols that activate the Nrf2 pathway [122], which increases the expression of hepcidin [11]. Furthermore, oats are rich in melatonin [123] which, in mice treated with an intraperitoneal injection, has shown to induce the gene expression of hepcidin in the liver and in serum levels through the activation of the transcriptional protein c-Jun [94]. Finally, the different concentrations and combinations of polyphenols in a food determine the effect on the release of hepcidin. Some polyphenols increase hepcidin levels, while other compounds reduce its concentration. For example, in Zebrafish embryos and in human HepG2 cells, quercetin treatment enhanced the level of hepcidin [95], and similar results were found in rats with quercetin [96]. Conversely, epigallocatenin3-gallate and myricetin led to a decrease in hepcidin levels in human and mouse hepatocytes [97] and in human hepatocellular carcinoma cells, in human embryonic kidney cells and in mice treated with LPS, respectively [98].

Some spices are also able to modulate hepcidin levels. Turmeric, in particular, showed important potential effects. In healthy male volunteers, the administration of 6 g of curcuma, corresponding to 120 mg of curcumin, reduced the serum levels of hepcidin for up to 24 h, probably through the inhibition of STAT3 action [99]. Additionally, a study conducted on male Wistar rats demonstrated that garlic increased plasma iron by inducing the expression of ferroportin, as well as decreased hepcidin levels in liver [100]. In fact, allicin, contained in garlic, may stimulate sirtuin-1 [124], which in turn suppresses the expression of hepcidin [35]. On the contrary, the consumption of chili pepper, whose main important component is capsaicin, may not be indicated in patients with anemia with increased hepcidin. In this context, one recent study reported that the chronic administration of capsaicin for 12 weeks increased hepcidin levels in the serum of diabetic mice [101].

The dietary intake of some vitamins can influence iron homeostasis through hepcidin. The importance of vitamin C in the treatment of anemia is known, since it increases the intestinal absorption of iron [88]. Even when not taken at the same time as foods rich in iron, vitamin C improves iron status in women [125]. It is plausible to think that this effect is mediated by the modulation of hepcidin. In fact, vitamin C inhibited hepcidin expression in human hepatocarcinoma cells [102] and reduced the levels of IL-6 in healthy persons following acute exercise [126]. 

Additionally, the effects of vitamin D on hepcidin have been extensively investigated. For example, in in vitro (human hepatocellular carcinoma and PBMC monocytes) and in vivo (mice and healthy volunteers) models, vitamin D administration inhibited hepcidin expression by binding to the gene promoter [103] and by reducing the levels of IL-6 and IL-1β in macrophages treated with LPS and in patients affected by chronic kidney disease [104]. Furthermore, a recent study reported the role of vitamin D in contrasting ferroptosis in Zebrafish liver cells [105]. Multiple observational studies report the association between vitamin D deficiency, high hepcidin levels and anemia in conditions characterized by inflammatory processes such as inflammatory bowel disease, acute infectious disease, severe traumatic injury and healthy preterm infants [127,128,129,130], while in other cases, vitamin D and iron deficiencies did not correlate with high hepcidin levels, even in the presence of inflammation [131]. Furthermore, in a study of pregnant women, vitamin D supplementation had no effect on hepcidin, ferritin and inflammatory status [106], however, the adequate intake of vitamin D during pregnancy correlated with better hemoglobin levels [132]. High-dose vitamin D supplementation improved iron status but did not affect hepcidin levels in athletes [107] nor in patients affected by chronic kidney disease [108]. Therefore, the impact of vitamin D supplementation on iron homeostasis and hepcidin concentrations needs to be further investigated.

Regarding Vitamin A, its deficiency has been correlated with increased hepcidin levels in rats [109]. In accordance with this, the administration of retinoic acid in mice with inflammatory anemia improved anemia by reducing hepcidin and increasing ferroportin [110]. However, in the absence of inflammation, the relationship between vitamin A and hepcidin seems less important, as reported in young male Wistar rats [111].

Vitamin E also plays a role in the expression of hepcidin: a recent study demonstrated that a diet rich in vitamin E increased the expression of ferroportin in mice and reduced hepcidin, through the suppression of the Nrf2 factor [112]. Understanding the role of minerals in iron metabolism is just beginning, and further studies are needed.

The role of carbohydrates in regulating hepcidin release is still controversial. Studies on carbohydrate supplementation before, during and after physical activity reported contradictory results on hepcidin levels [113,114,115]. In general, a low glycemic index diet is associated with low hepcidin levels [93], an effect probably linked to the reduced inflammatory state [133]. A high consumption of dietary fibers is also correlated with a decrease in hepcidin [93], perhaps due to an improvement in the microbiota, which counteracts inflammation. In this context, a study conducted on patients with renal anemia reported an improvement in anemia following the administration of dietary fiber, but without any significant effect on hepcidin [116].

Finally, colostrum of animal origin can be a valid supplement to improve anemic states characterized by an increase in hepcidin. A study demonstrated that a 6-month supplementation of bovine colostrum improved iron homeostasis in female athletes. The antioxidant action of colostrum reduced the levels of IL-6 and hepcidin in the group receiving the supplement compared to the control. Hepcidin reduction improved the availability of iron from the recycling system [117].

However, foods and nutrients that promote intestinal iron absorption, such as vitamin C, could increase hepcidin release following the increase in iron in the body, exhibiting a secondary modulation. Future studies are needed to ascertain this possible secondary action of foods on hepcidin, and the impact on anemias.

## 6. Conclusions

In the nutritional treatment of anemic states characterized by an increase in hepcidin, the sole evaluation of iron intake, through diet or supplements, may be ineffective or potentially dangerous due to ferroptosis. Conversely, considering hepcidin levels may be beneficial. These patients generally have high iron stores in the body. The reduction in hepcidin can improve the mobilization of accumulated iron, as well as promote the absorption of dietary iron. Similarly, when the increase in hepcidin is accompanied by iron deficiency, as in athletes, the decrease in hepcidin release increases the availability of iron which comes from erythrophagocytosis and intestinal absorption. In both cases, the reduction in hepcidin improves erythropoiesis, and counteracts the accumulation of intracellular iron, preventing ferroptosis. Although knowledge about the nutritional modulation of hepcidin is still at its early stages, it may offer interesting insights for nutritionists and physicians, so further studies are strongly needed in order to gain further insights about the effects of hepcidin on human health.

## Figures and Tables

**Table 1:** Known nutritional modulation of hepcidin. EPA, Eicosapentaenoic acid; DHA, Decosahexaenoic acid; IL-6, Interleukin 6; IL-1β, Interleukin 1 β; STAT3, Signal Transducer and Activator of Transcription 3; Nrf-2, Nuclear factor erythroid 2-related factor 2; c-Jun, protein c-Jun; GPX, glutathione peroxidase.

Foods	Experimental Model	Dose and Duration	Effects on Hepcidin Release	Proposed Mechanisms	Reference
Omega-3					
EPA/DHA	Mycobacterium tuberculosis-infected C3HeB/FeJ mice	AIN-93G control diet supplemented with EPA (44% of total fatty acids) and DHA (28% of total fatty acids) for 3 weeks	Decrease in hepcidin levels in plasma	Reduction in IL-1 and IL-6	[89]
Polyphenols-enriched foods and isolated polyphenols					
Caffeine	2-month-old C57BL/6N mice	Daily intragastric administration of caffeine (100 mg/kg body weight) for 7 days	Decrease in hepcidin levels in liver	Reduction in IL-6/STAT3	[90]
Wine	Patients with type 2 diabetes	300 mL of red wine daily for 3 weeks	Decrease in hepcidin levels in plasma	n.d.	[91]
Tucum-do-cerrado (*Bactris setosa* Mart.)	Wistar rats	AIN-93G diet supplemented with 150 g of the edible parts of the tucum-do-cerrado fruit/kg of diet for 12 weeks	Decrease in hepcidin levels in liver	Increase in Sirtuin-1	[92]
Dark leafy vegetables	Pregnant women	n.a.	Increase in hepcidin levels in serum	n.d.	[93]
Melatonin	8-week-old C57BL/6J mice	Intraperitoneal injection of melatonin (10 mg/kg)	Increase in hepcidin levels in serum Increase in hepcidin gene expression in liver	Increase in c-Jun pathway	[94]
Genistein	Zebrafish embryos Human hepatocellular carcinoma cells	7 µM from 28 to 52 for Zebrafish embryos, 0–20 µM for HepG2 cells	Increase in hepcidin levels	Increase in STAT3 and SMAD4	[95]
Quercetin	Male Sprague Dawley rats	Gavage or intraperitoneal injection (50 mg/kg) for 5 h or 10 days	Increase in hepcidin levels in liver	n.d.	[96]
Epigallocatenin3-gallate	Human and mouse hepatocytes	Treatment (100–200 ng)/intraperitoneal injection of epigallocatenin3-gallate (100 µM)	Decrease in hepcidin levels	Induction of small heterodimer partner-interacting leucine zipper protein	[97]
Myricetin	Human hepatocellular carcinoma cells, human embryonic kidney cells, Male C57BL/6 mice	20 µg/mL for 12 h or intraperitoneal injection of quercetin (40 mL/kg)	Decrease in hepcidin levels	Modulation of BMP/SMAD signaling	[98]
Spices					
Curcuma	Healthy male volunteers	6 g (corresponding to 120 mg of curcumin) for 0.5–48 h	Decrease in hepcidin levels in plasma	n.d.	[99]
Garlic	Male Wistar rats	Gavage (1 g/kg body weight) for 3 weeks	Decrease in hepcidin levels in liver	Increase in Sirtuin-1	[100]
Capsaicin	Male Wistar rats treated with streptozotocin to induce diabetes	Daily subcutaneous injection (1 mg/kg) for 12 weeks	Increase in hepcidin levels in liver	n.d.	[101]
Vitamins					
Vitamin C	Human hepatocellular carcinoma cells	50–100 µg/mL for 6 h	Decrease in hepcidin levels	n.d.	[102]
Vitamin D	Human hepatocellular carcinoma PBMC monocytes Male C57BL/6 mice Healthy volunteers	5 nM for 6 h Single intraperitoneal injections (0.2 μg/g) Single dose of oral vitamin D2 (100,000 IU).	Decrease in hepcidin levels	Transcriptional suppression of hepcidin gene	[103]
	THP-1 macrophage-like monocytic cells Patients affected by chronic kidney disease	5–40 nM overnight 50,000 IU weekly for 12 weeks	Decrease in hepcidin levels	Reduction in IL-1 and IL-6	[104]
	Zebrafish liver cells	200 pM for 72 h	Decrease in hepcidin levels	Inhibition of ferroptosis and modulation of Keap1–Nrf2–GPX4 and NF-κB–pathways	[105]
	Pregnant women	1000 IU daily for 14 weeks	Decrease in hepcidin levels in plasma	n.d.	[106]
	Ultra-marathon runner	10,000 UI daily for 2 weeks	No significant variation in hepcicin levels	n.d.	[107]
	Patients affected by chronic kidney disease	8000 IU of cholecalciferol daily for 12 weeks	No significant variation in hepcicin levels	n.d.	[108]
Vitamin A	Wistar rats	AIN-93G diet with or without 4000 IU/kg of diet for 57 days	Increase in hepcidin levels in deficient animals	n.d.	[109]
	BALB/c mice	3 or 15 mg/kg of retinoic acid for 14 days	Decrease in hepcidin levels	Modulation of TLT-4/NF-κB–pathways	[110]
	Young male Wistar rats	6 weeks	No significant variation in hepcicin levels	n.d.	[111]
Vitamin E	C57Bl/6 male mice	450 mg/kg for 18 days	Decrease in hepcidin levels in plasma	Reduction in Nrf2 pathway	[112]
Carbohydrates and fiber					
Complex carbohydrates	Endurance athletes	3–10 g/kg	Increase in hepcidin levels in serum	Increase in IL-6	[113]
	Endurance athletes	1.2 g/kg beverage (12 mL/kg, 10% carbohydrate beverage)	Increase in hepcidin levels in serum	Increase in IL-6	[114]
	Endurance athletes	3–8 g/kg	Variations time-dependent	Increase in IL-6	[115]
Dietary fiber	Patients affected by end-stage renal disease	10 g daily of dietary fiber or potato starch for 8 weeks	No significant variation in hepcicin levels	n.d	[116]
Supplements					
Bovine colostrum	Highly trained athletes	3.2 g (four capsules) daily for 6 months	Decrease in hepcidin levels in serum	Increase in IL-6	[117]

n.a.: Not available; n.d.: not determined.

## Data Availability

Not applicable.
